# Be Careful Where You Smile: Culture Shapes Judgments of Intelligence and Honesty of Smiling Individuals

**DOI:** 10.1007/s10919-015-0226-4

**Published:** 2015-12-30

**Authors:** Kuba Krys, C. -Melanie Vauclair, Colin A. Capaldi, Vivian Miu-Chi Lun, Michael Harris Bond, Alejandra Domínguez-Espinosa, Claudio Torres, Ottmar V. Lipp, L. Sam S. Manickam, Cai Xing, Radka Antalíková, Vassilis Pavlopoulos, Julien Teyssier, Taekyun Hur, Karolina Hansen, Piotr Szarota, Ramadan A. Ahmed, Eleonora Burtceva, Ana Chkhaidze, Enila Cenko, Patrick Denoux, Márta Fülöp, Arif Hassan, David O. Igbokwe, İdil Işık, Gwatirera Javangwe, María Malbran, Fridanna Maricchiolo, Hera Mikarsa, Lynden K. Miles, Martin Nader, Joonha Park, Muhammad Rizwan, Radwa Salem, Beate Schwarz, Irfana Shah, Chien-Ru Sun, Wijnand van Tilburg, Wolfgang Wagner, Ryan Wise, Angela Arriola Yu

**Affiliations:** Institute of Psychology, Polish Academy of Sciences, Jaracza 1, 00-378 Warsaw, Poland; Instituto Universitário de Lisboa (ISCTE-IUL), Cis-IUL, Lisbon, Portugal; Department of Psychology, Carleton University, Ottawa, Canada; Department of Applied Psychology, Lingnan University, Hong Kong, Hong Kong; Department of Management and Marketing, Faculty of Business, Hong Kong Polytechnic University, Hong Kong, Hong Kong; Psychology Department, Iberoamerican University, Mexico City, Mexico; Institute of Psychology, University of Brasilia, Brasília, Brazil; School of Psychology and Speech Pathology, Curtin University, Perth, Australia; Department of Psychiatry, JSS University, Karnataka, India; Department of Psychology, Renmin University of China, Beijing, China; Department of Communication and Psychology, Aalborg University, Aalborg, Denmark; Department of Psychology, University of Athens, Attica, Greece; Département Clinique du Sujet, Université Toulouse Jean Jaurès, Toulouse, France; Department of Psychology, Korea University, Seoul, Republic of Korea; Faculty of Psychology, University of Warsaw, Warsaw, Poland; Faculty of Arts, Menoufia University, Al Minufya, Egypt; Faculty of Sociology, Saint-Petersburg State University, St. Petersburg, Russia; Institute of Cognitive Neuroscience, Agricultural University of Georgia, Tbilisi, Georgia; University of New York Tirana, Tirana, Albania; Institute for Cognitive Neuroscience and Psychology, Hungarian Academy of Sciences, Budapest, Hungary; Department of Business Administration, International Islamic University Malaysia, Kuala Lumpur, Malaysia; College of Leadership Development Studies, Covenant University, Canaanland, Ota, Ogun State Nigeria; Istanbul Bilgi University, Istanbul, Turkey; Department of Psychology, University of Zimbabwe, Harare, Zimbabwe; Facultad de Humanidades y Ciencias de la Educación, Universidad Nacional de La Plata, La Plata, Argentina; Department of Education, University of Roma Tre, Rome, Italy; Faculty of Psychology, University of Indonesia, Depok, Indonesia; School of Psychology, University of Aberdeen, Aberdeen, UK; Department of Psychological Studies, Universidad ICESI, Cali, Colombia; Nagoya University of Commerce and Business, Nagoya, Japan; Department of Health Sciences and Health Policy, University of Luzern, Lucerne, Switzerland; Silver School of Social Work, New York University, New York City, NY USA; Department of Applied Psychology, Zurich University of Applied Sciences, Zurich, Switzerland; Department of Psychology, University of Sindh, Jamshoro, Pakistan; Department of Psychology, National Chengchi University, Taipei, Taiwan Republic of China; Department of Psychology, King’s College London, London, United Kingdom; Department of Social and Economic Psychology, Johannes Kepler University, Linz, Austria; Department of Psychology, University of the Philippines-Diliman, Quezon City, Philippines

**Keywords:** Smile, Honesty, Intelligence, Corruption, Uncertainty avoidance, Culture

## Abstract

Smiling individuals are usually perceived more favorably than non-smiling ones—they are judged as happier, more attractive, competent, and friendly. These seemingly clear and obvious consequences of smiling are assumed to be culturally universal, however most of the psychological research is carried out in WEIRD societies (Western, Educated, Industrialized, Rich, and Democratic) and the influence of culture on social perception of nonverbal behavior is still understudied. Here we show that a smiling individual may be judged as less intelligent than the same non-smiling individual in cultures low on the GLOBE’s uncertainty avoidance dimension. Furthermore, we show that corruption at the societal level may undermine the prosocial perception of smiling—in societies with high corruption indicators, trust toward smiling individuals is reduced. This research fosters understanding of the cultural framework surrounding nonverbal communication processes and reveals that in some cultures smiling may lead to negative attributions.

## Introduction

It is commonly recognized that it is good to smile—Louis Armstrong sang that when you smile the world smiles with you, and various trainers and guidebooks advise smiling because it improves interpersonal communication. These lay beliefs are supported by numerous studies demonstrating that smiling individuals are perceived as happier (Otta et al. 1994), more attractive, communal, competent (Hess et al. 2002; Matsumoto and Kudoh 1993), likable (Palmer and Simmons 1995), approachable, and friendly, and that a smile from another promises a safe and satisfying interaction (Miles 2009).

Cultures may shape different scripts for social behavior and as a consequence, different logics of nonverbal behavior and its social perception (Matsumoto 2006; Leung and Cohen 2011). In the past few decades, increasingly more psychological research has been carried out in non-WEIRD (Western, Educated, Industrialized, Rich, and Democratic; Henrich et al. 2010) societies indicating difficulties to replicate results from psychological experiments across cultures (Smith et al. 2013). Although psychologists broadly recognize the interrelationship between culture and behavior, and the sub-discipline called cross-cultural psychology is flourishing, interactions between culture and social perception of nonverbal behavior still remain understudied. One example of this lack of cross-cultural study is the assessment of cultural variation of traits attributed to smiling individuals that goes beyond East–West cultural comparisons (Hess et al. 2000). Rychlowska and collaborators (2015) were among the first to address this pointing to the importance of heterogeneity versus homogeneity of cultures in predicting the endorsement of smiling.

Although numerous studies confirm that positive perceptions of smiling individuals seem to be universal, anecdotal evidence suggests that in some cultures the opposite may be true. For example, a well-known Russian proverb says ‘Улыбкa, бeз пpичины - пpизнaк дypaчины’ (*smiling with no reason is a sign of stupidity*). The Norwegian government humorously explains nuances of Norwegian culture by indicating that when a stranger on the street smiles at Norwegians, they may assume that the stranger is insane (EURES 2010). British authors of a popular guidebook about Poland warn tourists that smiling at strangers is perceived by Poles as a sign of stupidity (Bedford et al. 2008). Even Darwin (1872/1998) wrote about “the large class of idiots who are … constantly smiling” (p. 199).

Previous studies have tested this counterintuitive phenomenon in different countries (Krys et al. 2014, 2015). However, these studies included only a small number of cultures (seven) compared to the much broader cross-cultural experiment reported here, which was conducted in 44 cultures. Cross-cultural comparisons involving that many different cultures allow for multilevel and country-level analyses, and are necessary to reliably identify cultural factors that are related to the differential social perception of the most often encountered facial expression, viz., the smile.

### Meanings Attributed to Smiles

Smiles are highly diverse in their types and in their possible meanings. They are used to communicate a range of different psychological signals, including positive emotions, social intentions, or a person’s social status (Matsumoto and Willingham 2009). Past research has offered a number of distinctions among smiles. The utility of one of the most popular distinctions, viz. Duchenne versus non-Duchenne smiles (Duchenne 1862), has been recently questioned because there is evidence that the use of the Duchenne marker of a ‘true’ smile is not universal, but rather limited to certain cultures (Abe et al. 2002; Thibault et al. 2012). In their simulation of smiles model, Niedenthal et nl. (2010) focus on the perception of smiles and suggest that the distinction between Duchenne and non-Duchenne smiles may be largely superseded by a distinction based on the functions of smiles, which may be derived from (and mapped onto) identifiable brain systems that represent different meanings of smiling.

Niedenthal et al. (2010) describe three types of smiles that have important and discrete functions, namely, enjoyment, affiliative, and dominance smiles. Humans (and some other primates) smile spontaneously during experiences of pleasure or success (Ekman 2009) and this expression is called the enjoyment smile. Affiliative smiles are those that signal positive social intentions and are essential for the creation and maintenance of social bonds; personal enjoyment does not have to accompany affiliative smiles. The third group of smiles—dominance smiles—reflect social status or control, and may include scheming smiles, critical smiles, and proud smiles which have different physical attributes than affiliative and enjoyment smiles (Niedenthal et al. 2010). Chang and Vermeulen (2010) claim that affiliative and enjoyment smiles cannot be discriminated from each other on the basis of physical markers—their meaning may be derived only from contextual information. Rychlowska and collaborators (2015) delivered evidence that the above distinctions may be cross-culturally identified, and documented that heterogeneity and homogeneity of cultures (i.e., the extent to which a country’s present-day population descended from migration from numerous vs. few source countries over a long period of time) may predict the endorsement of affiliative and dominance smiles, respectively.

The present research focuses on the attributions given to affiliative and enjoyment smiles presented in still photographs in order to uncover the cultural variation of meanings attributed to the most commonly expressed smiles. Limiting the scope of the current research in this way avoids the problems related to differences in cultural scripts that may influence the attributions to dominance smiles. We examined perceptions of honesty and intelligence attributed to smiling individuals because these traits reflect the big two of social perception (Abele and Wojciszke 2013; Bakan 1966). Among academic psychologists, there seems to be a consensus about two fundamental dimensions of social judgments, though these basic dimensions are named differently and have slightly different meanings. For example, Abele and Wojciszke (2014) call them agency and communion, whereas Fiske et al. (2006) use the labels warmth and competence. These dimensions reflect the logic of evolutionary pressure and help us determine whether ‘others’ are friend or foe (communion/warmth/honesty) and whether ‘others’ have the ability to enact their friendly or hostile intentions (agency/competence/intelligence).

### Cultural Predictors of Smile Perception

Descriptive accounts of general cultural differences have been available for a long time, but empirical assessments of cross-cultural variability have only emerged relatively recently (Hofstede 2001; House et al. 2004; Leung and Bond 2004). In our research we tested two predictions related to cultural variation. First, we tested the relation between cultural uncertainty avoidance (UA; House et al. 2004) and the social perception of smiling versus non-smiling individuals with regards to intelligence. Societies that rank high on UA socialize their members to alleviate the unpredictability of future events, whereas in societies that rank low on UA, the future is judged to be relatively unpredictable and there are fewer societal guidelines on how to behave (House et al. 2004). As argued elsewhere (Krys et al. 2014), in cultures low on UA, social conditions are regarded as uncertain, so expressing certainty through smiling (Hareli and Hess 2010) can be perceived as inconsistent behavior and people who exhibit inconsistency may be evaluated as unintelligent (Weisbuch et al. 2010).

The second hypothesis tests whether ‘corruption corrupts smiling’. We predicted that the more corrupt a society is, the less trust should be granted to a smile. On the one hand, a smile is the most common signal of positive intentions. In fact, a smile conveys a message that even a newborn baby understands and infants start smiling as early as 3 months old (Wörmann et al. 2014). The smile is perhaps the most commonly observed affiliative signal (Méhu and Dunbar 2008). A smile facilitates the establishment and maintenance of social bonds, and helps to coordinate social interactions (Fridlund 2002). All the above suggest that smiling evolved as a universal signal of honesty and functions as a social glue (Centorrino et al. 2015). On the other hand, this social glue may be counterfeited without difficulty because smiling is a signal that can be easily produced (Méhu 2011). In particular circumstances, some smiles may be expressed to benefit the signaller and may be deceptive (Ekman and Friesen 1982).

Therefore, we predicted that the ease of producing a smile may in some conditions lead to lower trust in this signal and one of the pre-conditions of scepticism about a smile’s honesty is excessive corruption in society. In highly corrupt societies, individuals are exposed to relatively frequent unfair or untruthful behaviors and, thus, scepticism about the positive intentions underlying a smile may be well-grounded and justified. Hence, in our second hypothesis we offer the novel prediction that the higher the corruption index of a country, the more smiling individuals will be perceived as dishonest. In other words, we empirically tested whether ‘corruption corrupts’ the evolutionary social glue of the smile.

Past research has shown that social judgements of smiling and non-smiling individuals may also be affected by gender-related expectations (Hess et al. 2009). Gender stereotypes and beliefs about emotional expressiveness can lead to different standards when men and women evaluate the nonverbal behavior of other men and women (Krumhuber et al. 2007). Women tend to smile more than men (LaFrance et al. 2003; Hall 1984) and there is a greater expectation for them to do so (Brody and Hall 2008). Therefore, the gender of the assessor and poser were included as control variables in all analyses. The contributions of these control variables will be reported, though a detailed discussion of this contribution is beyond the scope of the current paper.

## Method

To provide a systematic analysis of the social perception of smiling individuals, we asked participants in 44 cultures to rate photos of smiling and non-smiling individuals on traits assessing honesty and intelligence.

### Participants and Selection of Cultures

Data were gathered from a total of 5216 respondents in 44 cultures across six continents. After removing individuals with at least one missing answer on the measures of intelligence or honesty, the final sample that was analysed consisted of 4519 participants. In Table 1, we present demographic characteristics for all national samples. The gender distribution was 56.5 % female and 43.5 % male. The mean age of participants was 22.36 years (*SD* 5.50). Participants were students from a variety of different disciplines who were recruited at each author’s university. All data were collected from 2011 to 2015.

**Table 1:** Samples’ characteristics

	coll.	anlz.	fem.	fem.	age	age	intell.	hones.	intell.	hones.
	*N*	*N*	*N*	%	*M*	*SD*	*α*	*α*	*d*	*d*
Albania	119	90	50	56	20.81	2.05	.81	.69	.14	.44
Argentina	104	80	51	64	33.77	10.53	.76	.73	.04	−.03
Australia	120	112	86	77	19.74	3.93	.91	.83	.24	.70
Austria	109	95	60	63	25.57	6.35	.92	.88	.49	.56
Brazil	120	103	68	66	23.89	5.70	.77	.71	.31	.47
Canada	117	86	49	57	20.34	4.05	.92	.88	.20	.56
China	120	111	51	46	23.09	4.10	.84	.85	.54	.53
Colombia	120	113	70	62	27.66	13.46	.81	.75	.05	.69
Denmark	112	106	53	50	23.61	2.90	.91	.81	.31	.51
Egypt	93	61	49	80	20.79	4.45	.85	.76	.37	.53
France	120	102	61	60	28.19	8.77	.90	.86	−.16	.23
Georgia	120	115	58	50	25.30	10.06	.70	.69	.22	.54
Germany	81	78	36	46	22.71	6.02	.84	.76	1.01	.43
Greece	125	120	62	52	20.82	1.59	.85	.76	−.04	.62
Hong Kong	120	112	49	44	20.44	1.77	.68	.76	.01	.26
Hungary	118	105	57	54	21.28	3.79	.86	.78	.00	.41
India Karnataka	120	92	49	53	21.15	2.77	.83	.50	−.03	−.08
India Kerala	120	104	54	52	20.32	1.26	.79	.62	−.41	.03
Indonesia	120	120	60	50	19.58	1.37	.75	.69	.09	.00
Iran	48	42	31	74	21.21	4.08	.79	.70	−.40	.09
Ireland	120	104	46	44	19.35	3.08	.88	.80	.14	.51
Israel	99	83	31	37	26.24	5.15	.87	.84	−.12	.26
Italy	160	151	137	91	23.12	6.14	.82	.79	.01	.56
Japan	109	103	52	51	19.24	1.21	.82	.83	−.41	.47
Kuwait	300	298	132	44	21.46	3.22	.71	.62	.12	.31
Malaysia	120	100	73	73	22.81	4.34	.88	.80	.57	.55
Maldives	120	95	44	46	24.09	2.99	.78	.55	.08	−.01
Mexico	136	105	54	51	21.07	2.34	.91	.82	−.09	.35
Nigeria	120	112	59	53	19.16	1.56	.83	.70	.25	.35
Norway	97	85	50	59	22.22	3.76	.87	.70	.05	.41
Pakistan	190	137	68	50	21.52	3.22	.78	.63	.26	.19
Philippines	120	114	83	73	19.22	2.00	.83	.78	.35	.70
Poland	76	68	48	71	22.69	1.81	.90	.79	−.02	.43
Portugal	120	111	62	56	22.03	3.15	.79	.67	.25	.61
Russia	120	113	71	63	22.33	1.87	.87	.79	−.28	.21
So. Afr. n-white	115	41	24	59	20.93	1.82	.83	.76	−.02	.46
So. Afr. white	115	43	21	49	20.93	1.82	.83	.76	.10	.51
South Korea	120	112	62	55	20.81	2.25	.74	.70	−.36	.52
Switzerland	107	99	59	60	25.04	5.62	.92	.90	.96	.91
Taiwan	68	61	37	61	19.51	.94	.81	.78	.15	.48
Turkey	134	127	64	50	22.57	2.71	.76	.65	.22	.36
UK	120	111	59	53	23.82	8.99	.88	.80	.32	.62
USA	84	79	52	66	24.06	9.81	.94	.90	.15	.50
Zimbabwe	120	120	60	50	22.82	3.70	.66	.55	.16	.02
Average	119	103	58	57	22.44	4.15	.83	.75	.13	.40
Total	5216	4519	2552	56	22.36	5.50	.85	.79	.12	.39

*coll. N* N collected, *anlz. N* N analyzed (all further data are presented for N analyzed); *fem. N* N female, *fem. %* percentage of female in a sample; *intell. α* Cronbach’s alpha for intelligence measure, *hones. α* Cronbach’s alpha for honesty measure, *So. Afr. n-white/white* South Africa non-white/white samples (for South Africa we follow the GLOBE distinction)

We managed to collect data in forty-two out of the sixty-two cultures involved in the GLOBE project (House et al. 2004). We also collected data in Norway (for which practices on the GLOBE dimensions were calculated by Warner-Søderholm 2010) and in Pakistan (ranked high on the corruption dimensions, which relates to our second hypothesis). We aimed to collect data from at least 120 individuals in each analyzed culture (some authors, however, collected more and other authors collected fewer).

## Materials

**Fig. 1:**
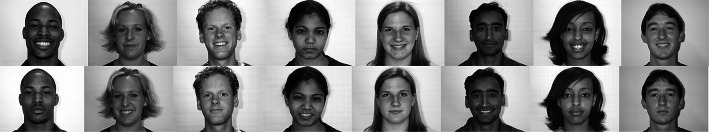
Photographs used in the current study. Participants assessed either the faces in the *upper* or those in the *lower row*

All participants were asked to provide basic demographic information on their gender, age, student status, religion, and father’s highest degree. Individuals were also asked about their ethnicity and nationality in cultures where the team leaders decided that asking about this information was not controversial. The main part of the questionnaire had participants rate eight faces, four smiling and four non-smiling, that were balanced for gender and represented different ethnicities (four European American, two African American, and two Indian, see Fig. 1; the need for ethnic diversity is stressed by Matsumoto and Kudoh 1993) on a 7-point Likert-type scale (1 = *trait doesn’t fit at all* to 7 = *trait fits perfectly*) measuring intelligence (i.e., *intelligent*, *dumb*, *smart*, and *stupid*) and honesty (i.e., *honest*, *false*, *authentic*, and *unnatural*). Questionnaires in cultures that joined the project later also included five items from Rosenberg’s self-esteem scale (1965), three items from Dunton and Fazio’s motivation to control prejudiced reactions scale (1997), and three additional attributes (i.e., *attractiveness*, *friendliness*, and *familiarity*) using the aforementioned 7-point scale. The current report is of the data provided by all participants. Photographs of the same persons posing neutral and smiling expressions were taken from the Center for Vital Longevity Face Database (Minear and Park 2004). The questionnaire, in the form of a small booklet, started with the following instructions: “Research shows that people can quite accurately evaluate others based on their looks. Can you help us and rate some faces?”. Photographs were organised into two sets, with targets who were smiling in one set presented as non-smiling in the other (Fig. 1). Half of the participants received one set, the other half received the other set. Photographs in each set were randomized. In a pre-test carried out among 183 Polish students, smiling faces, were assessed as more joyful, *t*(173) = 18.43, *p* < .001, *d* = 1.40, and affiliative, *t*(182) = 9.22, *p* < .001, *d* = .68, than non-smiling faces, but did not differ in dominance, *t*(176) = .25, *p* = .80, *d* = .02. Materials were originally written in Polish and English and were translated from English into languages of each country where the study was carried out. Following best practices (Brislin 1970), team leaders in each culture were asked to follow the back-translation procedure to establish linguistic equivalence. The original material, including the manual for collaborating researchers, is available from the first author in English.

For each participant, we calculated the average ratings given to smiling and non-smiling target individuals across the traits associated with the intelligence and honesty dimensions. Next, an effect size (i.e., Cohen’s *d*) for the differences between ratings for smiling and non-smiling individuals was calculated for each dimension in each culture. Thus, we obtained two measures for each culture: a Cohen’s *d* for intelligence and a Cohen’s *d* for honesty.

## Results

**Fig. 2:**
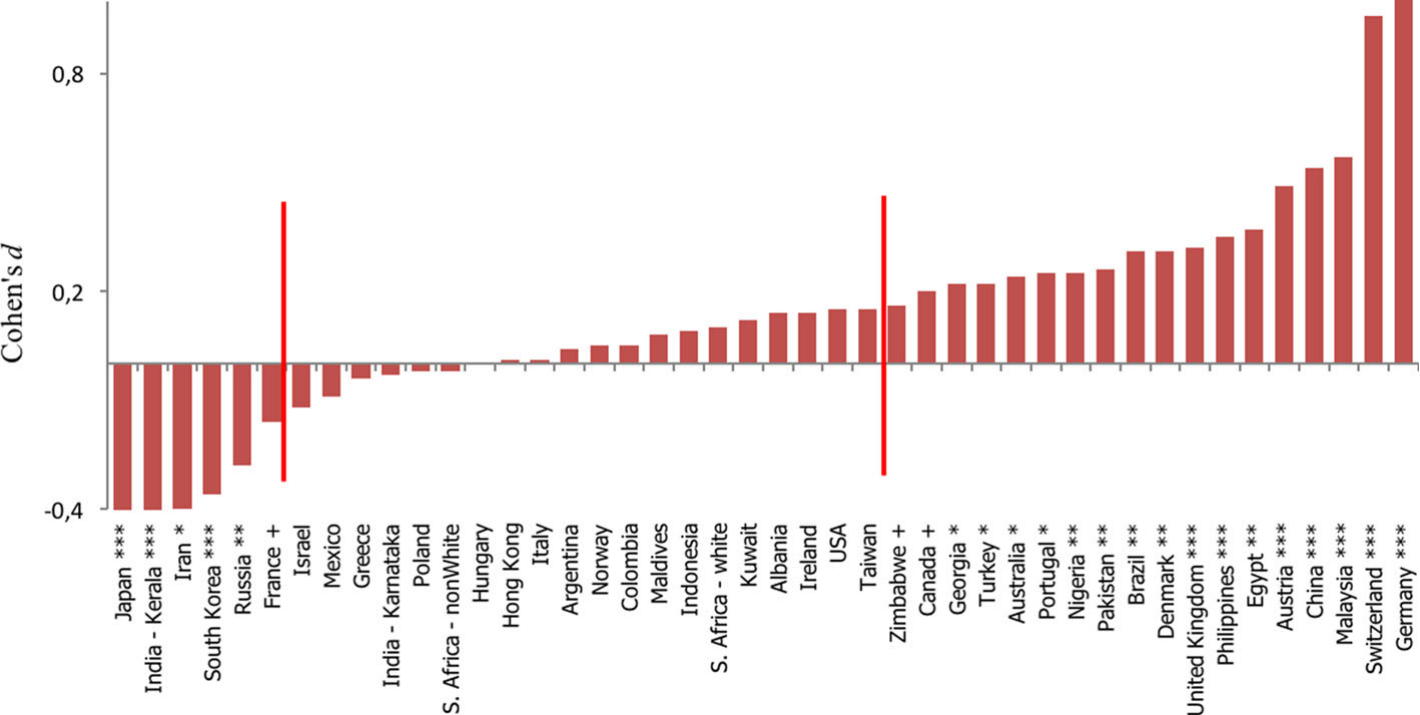
Cohen’s d for the difference in intelligence ratings of smiling and non-smiling individuals across cultures. *Red lines* separate cultures in which smiling individuals are rated as significantly more intelligent (on the *right*) or significantly less intelligent (on the *left*)

**Fig. 3:**
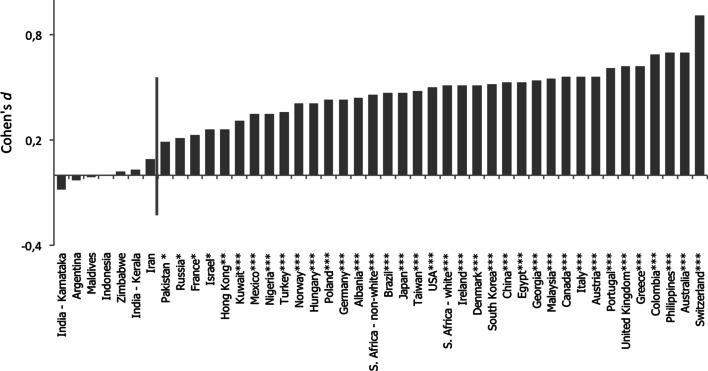
Cohen’s d for the difference in honesty ratings of smiling and non-smiling individuals across cultures. *Red line* separates cultures in which smiling individuals are rated as significantly more honest (on the *right*)

To test our two predictions, we separately examined cross-cultural differences in ratings given to smiling and non-smiling individuals on the intelligence and honesty dimensions. The results are summarised in Figs. 2 and 3.

As predicted, smiling was not consistently perceived as a signal of intelligence across all cultures. Although smiling did lead to attributions of higher intelligence in 18 out of the 44 cultures, we identified six cultures where individuals were perceived as significantly less intelligent when smiling. Moreover, we found no significant difference in the intelligence ratings of smiling versus non-smiling individuals in 20 cultures.

This result supports our prediction that social perception of the intelligence of smiling individuals varies across cultures, and in some cultures, smiling may even lead to attributions of lower levels of intelligence (we observed a significant two-way culture by smile interaction, *F*(43, 4425) = 5.9, *p* < .001, *η*_p_^2^ = .05; for detailed summary of results of ANOVA analyses, including gender of participant and gender of target, see Table 2). As predicted, the key to understanding this variability was not in geography (e.g., neighboring countries like China and Japan or Germany and France are on different ends of the distributions) nor in economic factors, but in cultural dimensions (for detailed correlation and regression analyses, including economic factors, at the cultural level examine Table 3). The predicted correlation between Cohen’s *d* (i.e., the difference in rated intelligence of smiling and non-smiling individuals) and UA practices was high, *r* = .63, *p* < .001, and remained strong after controlling for economic factors (*β* = .65, *p* < .001). UA practices remained a significant predictor even when controlling for the heterogeneity-homogeneity of 28 cultures in our study that had heterogeneity-homogeneity data available (Rychlowska et al. 2015; *β* = .67, *p* < .001). Consistent with predictions, multilevel analyses revealed a significant cross-level interaction between facial expression and UA in the prediction of intelligence attributions (for details of these analyses see Table 4).

**Table 2:** Results of two ANOVA analyses for intelligence and honesty perception

	Intelligence perception		Honesty perception		*df*
	*F*	*η squared*	*F*	*η squared*	
Smile	45.9***	**.010**	540.6***	**.109**	1, 4425
Culture	13.3***	**.115**	20.3***	**.165**	43, 4425
PG	94.2***	**.021**	50.2***	**.011**	1, 4425
TG	24.5***	**.006**	78.6***	**.017**	1, 4425
Smile × culture	5.9***	**.054**	4.5***	**.042**	43, 4425
Smile × PG	6.7**	**.002**	11.2**	**.003**	1, 4425
Smile × TG	1.5	.000	41.9***	**.009**	1, 4425
Culture × PG	3.9***	**.037**	2.3***	**.022**	43, 4425
Culture × TG	4.3***	**.040**	4.2***	**.039**	43, 4425
PG × TG	47.4***	**.011**	1.7	.000	1, 4425
Smile × culture × PG	.8	.008	1.3	.012	43, 4425
Smile × culture × TG	1.3	.013	1.0	.010	43, 4425
Smile × PG × TG	.5	.000	1.0	.000	1, 4425
Culture × PG × TG	1.7**	**.016**	1.1	.010	43, 4425
Smile × culture × PG × TG	1.1	.011	.6	.006	43, 4425

*PG* participant’s gender, *TG* target’s gender. Smile and gender of target as within-subjects factors, and culture and gender of observer as between-subjects factors. Significant values are shown in bold

** *p* < .01, *** *p* < .001

**Table 3:** Analysis at the cultural level: correlations and standardized regression coefficients

	Intelligence *d*	Honesty *d*
*Correlations (r)*		
Cultural practices (GLOBE project)		
Uncertainty avoidance	.63***	.24
Power distance	−.16	−.28
Institutional collectivism	−.14	.05
In-group collectivism	−.29	−.37*
Gender egalitarianism	−.06	.16
Assertiveness	.21	.20
Future orientation	.36*	.28
Performance orientation	.22	.11
Humane orientation	−.03	−.17
Cultural values (Schwartz—S; Hofstede—H)		
Harmony—S	.26	.28
Embeddedness—S	−.22	−.32*
Hierarchy—S	−.34*	−.35*
Mastery—S	−.22	−.21
Affective autonomy—S	.17	.18
Intellectual autonomy—S	.24	.36*
Egalitarianism—S	.32*	.35*
Power distance—H	−.21	−.25
Individualism—H	.13	.23
Masculinity—H	.09	.23
Uncertainty avoidance—H	−.30	.00
Long term orientation—H	.02	.05
Indulgence—H	.15	.34*
Social axioms (Bond et al. 2004)		
Dynamic externality	.06	−.36
Societal cynicism	−.13	−.29
Corruption indexes		
Corruption perception index—ranking	−.23	−.53***
Global corruption barometer—paying bribe	−.27	−.53**
Bribe payers index	.41	.59**
Economic freedom Index—corruption	.28	.51***
Socio-economic indexes		
GDP *per capita*	.21	.38*
GDP PPP	−.01	−.02
GINI index	−.01	−.07
Historical heterogeneity (vs. homogeneity)	.09	.29
Life expectation at birth	.09	.38*
Literacy rate	−.29	.23
Military expenditures (% GDP)	−.22	−.09
Population density	−.09	−.14
Population growth	.05	−.10
Rural population (% total)	−.02	−.32*
Unemployment rate	−.13	−.08
Regressions (*β*)		
	Model 1	
Uncertainty avoidance (GLOBE)	.65***	–
GDP *per capita*	.02	–
GINI Index	.15	–
		Model 2
Corruption perception index	–	−.48*
GDP *per capita*	–	−.02
Life expectation at birth	–	.03
Rural population (% total)	–	−.01

Highly significant (*p* < .01) values are shown in bold

* *p* < .05, ** *p* < .01, *** *p* < .001

**Table 4:** Unstandardized coefficients from multilevel linear regression analyses of perceived intelligence (Models 1 and 2) and perceived honesty (Models 3 and 4)

	Model 1	Model 2	Model 3	Model 4
	DV: perceived intelligence		DV: perceived honesty	
	IV culture: uncertainty avoidance		IV culture: Corruption Perceptions Index	
Intercept	4.7459 (0.2719)***	4.7399 (0.2557)***	4.6347 (0.0688)***	4.62484 (0.0659)***
Culture	0.0105 (0.0651)	0.0055 (0.0612)	−0.0007 (0.0008)	−0.0004 (0.0007)
Smile	−0.8997 (0.1684)***	−0.7745 (0.0907)***	0.3419 (0.0372)***	0.3233 (0.0226)***
PG	−0.8322 (0.2162)***	−0.5479 (0.1466)***	−0.1399 (0.0455)**	−0.1099 (0.0167)***
TG	−0.0153 (0.1684)	−0.0777 (0.0118)***	−0.1409 (0.0372)***	−0.1307 (0.0226)***
Culture × smile	0.2420 (0.0405)***	0.2096 (0.0217)***	−0.0014 (0.0005)**	−0.0017 (0.0003)***
Culture × PG	0.1489 (0.0520)**	0.0891 (0.0355)*	0.0009 (0.0006)	–
Smile × PG	0.2191 (0.2596)	–	−0.0255 (0.0560)	–
Culture × TG	−0.0349 (0.0405)	–	−0.0013 (0.0005)**	−0.0011 (0.0003)***
Smile × TG	0.1016 (0.2381)	–	0.1876 (0.0526)***	0.1780 (0.0225)***
PG × TG	0.4127 (0.2596)	–	0.0388 (0.0560)	–
Culture × smile × PG	−0.0698 (0.0621)	–	−0.0009 (0.0007)	–
Culture × smile × TG	−0.0146 (0.0572)	–	0.0002 (0.0007)	–
Culture × PG × TG	−0.0658 (0.0621)	–	0.0003 (0.0007)	–
Smile × PG × TG	−0.1263 (0.3671)	–	−0.0538 (0.0791)	–
Culture × smile × PG × TG	0.0322 (0.0878)	–	0.0001 (0.0010)	–

*PG* participant’s gender, *TG* target’s gender. For models 1 and 2, *culture* means culture-level predictor Uncertainty Avoidance; for models 3 and 4 *culture* means culture-level predictor Corruption Perceptions Index. Robust standard errors are given in parentheses

* *p* < .05, ** *p* < .01, *** *p* < .001

We also found support for our second hypothesis. Although smiling individuals were perceived as more honest than non-smiling individuals in almost all analyzed cultures (37 out of 44), there was cultural variability in the size of the effect (we observed a significant two-way culture by smile interaction, *F*(43, 4425) = 4.5, *p* < .001, *η*_p_^2^ = .04; for a summary of ANOVA analyses see Table 2). Moreover, this cultural variability was related to societal corruption levels (see Table 3). The correlation between a smile’s honesty bonus and three different corruption indices: Corruption Perceptions Index (Transparency International 2010a), Global Corruption Barometer—Paying Bribe sub-dimension (Transparency International 2010b), and Index of Economic Freedom – sub-index Freedom from Corruption (The Heritage Foundation 2010), was significant and relatively high (.59 > *r* > .51, *p* < .01) and remained significant after controlling for socio-economic factors (*β* = −.48, *p* = .04). Corruption remained a significant predictor even when controlling for the heterogeneity-homogeneity of 28 cultures in our study that had heterogeneity-homogeneity data available (Rychlowska et al. 2015; *β* = −.64, *p* < .001). Multilevel analyses revealed an interaction between facial expression and corruption index in the prediction of honesty judgments (see Table 4). As predicted, greater corruption levels decreased trust granted toward smiling individuals.

Beyond cultural variability, we found that participant and target gender were important factors that influenced the social perception of smiling versus non-smiling individuals (see Table 2). For intelligence perception, we observed a significant two-way participant gender by smile interaction, indicating that smiling increases ratings of intelligence more among women (*t*[2551] = 7.80, *p* < .001, *d* = .15, *M*_Non-smileFemale_ = 4.71, *SD*_Non-smileFemale_ = .75, *M*_SmileFemale_ = 4.83, *SD*_SmileFemale_ = .77) than among men (*t*[1960] = 3.37, *p* = .001, *d* = .08, *M*_Non-smileMale_ = 4.54, *SD*_Non-smileMale_ = .76, *M*_Smile_Male_ = 4.61, *SD*_Smile_Male_ = .78). Two remaining two-way interactions regarded perceptions of honesty. A significant participant gender by smile interaction indicated that smiling increases ratings of honesty more for female assessors (*t*[2551] = 22.66, *p* < .001, *d* = .45, *M*_Non-smileFemale_ = 4.48, *SD*_Non-smileFemale_ = .71, *M*_SmileFemale_ = 4.83, *SD*_SmileFemale_ = .73) than for male assessors (*t*[1960] = 13.73, *p* < .001, *d* = .31, *M*_Non-smileMale_ = 4.40, *SD*_Non-smileMale_ = .67, *M*_SmileMale_ = 4.64, *SD*_SmileMale_ = .72). Finally, a significant target gender by smile interaction revealed that non-smiling women were assessed as more honest than non-smiling men (*t*[4518] = 12.76, *p* < .001, *M*_Non-smile_Female_ = 4.55, *SD*_Non-smile_Female_ = .86, *M*_Non-smile_Male_ = 4.36, *SD*_Non-smile_Male_ = .89), but smiling men and women were found to be equally honest (*t*[4518] = 1.40, *p* = .16, *M*_Smile_Female_ = 4.77, *SD*_Smile_Female_ = .89, *M*_Smile_Male_ = 4.74, *SD*_Smile_Male_ = .91).

## Discussion

In sum, the data illustrate that the perception of smiling individuals is culturally diversified and that, in some cultures, this generally positive nonverbal signal may have negative associations. In addition, this research indicates that corruption at the societal level may weaken the meaning of an evolutionary important signal such as smiling and undermine its trustworthiness. Our results also show that the gender of the assessor and target have an important influence on the social perception of smiles, which may be related to socialization processes and strong stereotypical expectations for women to be more communal and to smile (LaFrance et al. 2003). Across cultures, smiling increased attributions of intelligence and honesty more for female assessors than for males and target gender affected attributions of honesty in non-smiling targets, but not for smiling targets. These effects of participant and target gender on smile perception did not affect the interactions of culture and smiling, however, which are the focus of the current report.

Although the results are statistically significant, causal inferences need to be drawn with caution because the relationships indicated in our research are mainly correlational. Another limitation of the presented research is that the samples may not be fully representative of the cultures they come from (i.e., predominantly university students were recruited to participate). Furthermore, the situational context, which was not manipulated here, may also play an important role in the perception and judgment of smiles (Niedenthal et al. 2010). Lastly, future studies are needed to examine the potential influence of participant ethnicity as attributions may differ across in-group and out-group faces. The role of smile intensity is also another ripe area for future research (see Kraus and Chen 2013 for instance).

Despite these limitations, this cross-cultural study illuminates surprising nuances of up-to-now seemingly clear and obvious processes of smile perception. Although numerous studies suggest that smiling individuals are perceived favorably, we document that the same person may be judged as less intelligent when smiling than when posing a neutral expression in some cultures. This has important practical implications, for example, in the context of globalization and job applications. In many countries it is still common to submit photographs on one’s CV. Knowing whether a smile is interpreted positively (i.e., as a sign of competence and trustworthiness) or negatively may be crucial knowledge for international applicants (also see Ruben et al. 2014).

Furthermore, this study advances theory about nonverbal behavior in important ways. By recognizing processes underlying the two cultural dimensions used in perceiving smiles, we indicate that those seemingly counter-intuitive findings reflect on highly functional strategies in their own cultural context (also see Matsumoto 2006). Expressing certainty in uncertain social conditions may not be the best way of signalling intelligence (Hypothesis 1), and signalling unconditional trust in untrustworthy settings may be risky (Hypothesis 2). Our research underscores the importance of the cultural framework in understanding nonverbal communication processes and reveals that although positive traits are usually attributed to smiling persons, perception of this common nonverbal signal may have unexpected negative implications in some cultures.
